# Intraoperative breakage of Sachse’s knife blade: a rare complication of optical internal urethrotomy (one case managing experience)

**DOI:** 10.1590/S1677-5538.IBJU.2016.0081

**Published:** 2017

**Authors:** Gautam Kumar Kanodia, Satyanarayan Sankhwar, Ankur Jhanwar, Ankur Bansal, Manoj Kumar, Ashok Gupta

**Affiliations:** 1 King George Medical University, Lucknow, Uttar Pradesh, India

**Keywords:** Urethra, Recurrence, Methods

## Abstract

Optical internal urethrotomy (OIU) is the most common procedure performed for short segment bulbar urethral stricture worldwide. This procedure most commonly performed using Sachse’s cold knife. Various perioperative complications of internal urethrotomy have been described in literature including bleeding, urinary tract infection, extravasation of fluid, incontinence, impotence, and recurrence of stricture. Here we report a unique complication of breakage of Sachse knife blade intraoperatively and its endoscopic management.

## INTRODUCTION

Optical internal urethrotomy (OIU) is the most common procedure performed for short segment bulbar urethral stricture worldwide (1). However, its success rate is variable, and ranges from 35-60% (2, 3). This procedure is most commonly performed using Sachse’s cold knife (4), although recently lasers have been introduced in the urological armamentarium for internal urethrotomy. Various perioperative complications of internal urethrotomy have been described in literature, including bleeding, urinary tract infection, extravasation of fluid, incontinence, impotence, and recurrence of stricture (5). Here we report a unique complication of breakage of Sachse knife blade intraoperatively and its endoscopic management.

### Case Report

**Figure 1:**
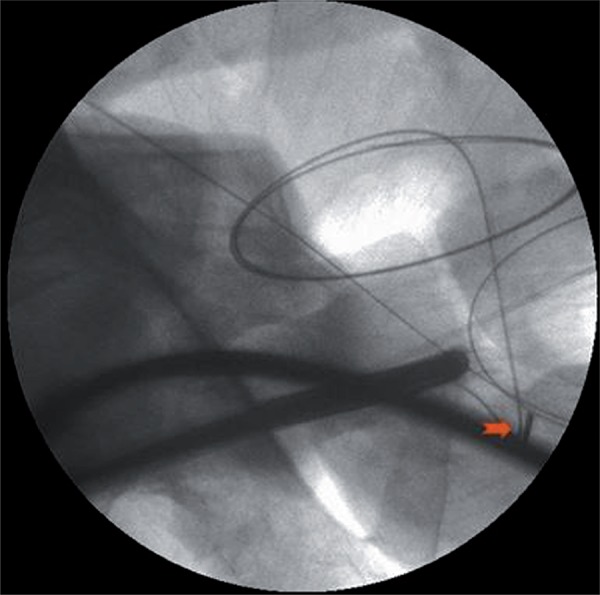
Fluroscopic view of broken blade in bulbar urethra.

**Figure 2:**
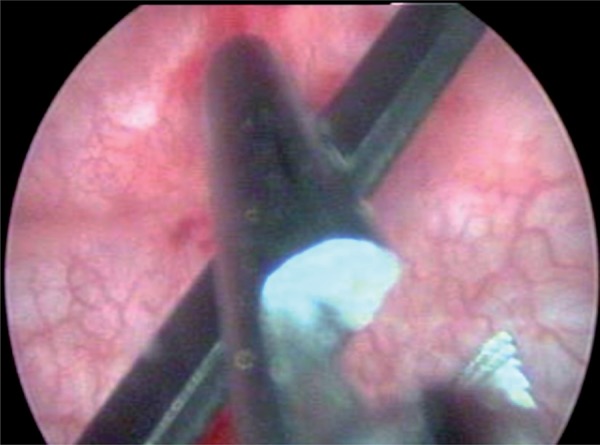
Cystoscopic view of holding broken blade with Double J removal forceps.

**Figure 3:**
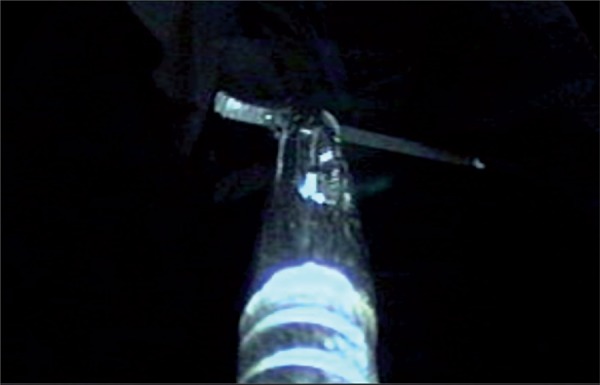
Removal of knife blade.

A 30 year-old male presented with complaint of lower urinary tract symptoms for the last six months. Uroflowmetry voiding pattern was suggestive of urethral stricture disease. Retrograde urethrography (RGU) revealed a short segment bulbar urethral stricture (<1.5cms). Optical internal urethrotomy was performed. Intraoperatively blade of Sachse’s urethrotome accidently broken and fell proximal to the stricture which was confirmed on fluoroscopy (Figure-1). We completed the procedure with another working element. During the procedure broken blade migrated to bladder (Figure-2). We retrieved the blade into the cystoscope sheath (22Fr) with the help of double J stent removing forceps. Cystoscope, sheath, forceps and the broken blade were withdrawn from the urethra as a single unit (Figure-3).

## DISCUSSION

Optical internal urethrotomy became popularized after the work of Sachse in 1971 (6) and now it is the preferred treatment modality for a short segment urethral stricture. This is the most favored procedure among the urologist as it is less morbid and minimally invasive day care surgery which is appealing to both patient and surgeon. The most common complications are recurrence of the stricture and bleeding (7, 8). The purpose of this case report is to highlight the unique complication of intraoperative breakage of knife blade and its endoscopic management. One should not start this (neither any other) procedure not being prepared to all its complications and that blade breakage is one of these, making necessary a blade backup and a double-J forceps available before starting this procedure. To the best of our knowledge, this is the only case report which describe this unique complication and management.

## CONCLUSIONS

This complication should be kept in mind and instruments should be checked properly by the operative surgeon prior to start the procedure. Retained sharp objects like knife blade in urethra as a result of breakage of Sachse knife blade can be managed endoscopically.
